# Navigating the Functional Landscape of Transcription Factors via Non-Negative Tensor Factorization Analysis of MEDLINE Abstracts

**DOI:** 10.3389/fbioe.2017.00048

**Published:** 2017-08-28

**Authors:** Sujoy Roy, Daqing Yun, Behrouz Madahian, Michael W. Berry, Lih-Yuan Deng, Daniel Goldowitz, Ramin Homayouni

**Affiliations:** ^1^Bioinformatics Program, University of Memphis, Memphis, TN, United States; ^2^Center for Translational Informatics, University of Memphis, Memphis, TN, United States; ^3^Computer and Information Sciences Program, Harrisburg University of Science and Technology, Harrisburg, PA, United States; ^4^Department of Mathematical Sciences, University of Memphis, Memphis, TN, United States; ^5^Department of Electrical Engineering and Computer Science, University of Tennessee, Knoxville, TN, United States; ^6^Center for Molecular Medicine and Therapeutics, University of British Columbia, Vancouver, BC, Canada; ^7^Department of Biological Sciences, University of Memphis, Memphis, TN, United States

**Keywords:** biomedical text mining, tensor factorization, tensor decomposition, multiway analysis, applied multilinear algebra, transcription factors

## Abstract

In this study, we developed and evaluated a novel text-mining approach, using non-negative tensor factorization (NTF), to simultaneously extract and functionally annotate transcriptional modules consisting of sets of genes, transcription factors (TFs), and terms from MEDLINE abstracts. A sparse 3-mode term × gene × TF tensor was constructed that contained weighted frequencies of 106,895 terms in 26,781 abstracts shared among 7,695 genes and 994 TFs. The tensor was decomposed into sub-tensors using non-negative tensor factorization (NTF) across 16 different approximation ranks. Dominant entries of each of 2,861 sub-tensors were extracted to form term–gene–TF annotated transcriptional modules (ATMs). More than 94% of the ATMs were found to be enriched in at least one KEGG pathway or GO category, suggesting that the ATMs are functionally relevant. One advantage of this method is that it can discover potentially new gene–TF associations from the literature. Using a set of microarray and ChIP-Seq datasets as gold standard, we show that the precision of our method for predicting gene–TF associations is significantly higher than chance. In addition, we demonstrate that the terms in each ATM can be used to suggest new GO classifications to genes and TFs. Taken together, our results indicate that NTF is useful for simultaneous extraction and functional annotation of transcriptional regulatory networks from unstructured text, as well as for literature based discovery. A web tool called Transcriptional Regulatory Modules Extracted from Literature (TREMEL), available at http://binf1.memphis.edu/tremel, was built to enable browsing and searching of ATMs.

## 1 Introduction

The complexity of organisms is correlated with the number of mechanisms by which gene expression is regulated in response to environmental and developmental signals (Levine and Tjian, 2003; Chen and Rajewsky, 2007; Davidson, 2010). Transcriptional regulation involves complex gene regulatory networks (GRNs), consisting of structural proteins involved in chromatin remodeling and transcription factors that regulate the core transcriptional machinery (Djebali et al., 2012). An active area of research is focused on integration of various high-throughput “omic” data in order to understand how genes are functionally regulated and involved in physiological and pathological processes (Gerstein et al., 2012). However, aggregation and annotation of gene regulatory networks (GRNs) from various sources remain challenging. Some GRN annotation is available in repositories such as KEGG (Kanehisa et al., 2004) and GO (Ashburner et al., 2000). However, these knowledge bases are incomplete and too general to provide specific insights into GRNs. More recent efforts have focused on manually integrating GRN information from various data sources (Liu et al., 2015). In addition, methods to automatically annotate GRNs based on semantic relationships in the biomedical literature are beginning to be developed (Chen et al., 2014). Currently, there are more than 23 million citations in MEDLINE, many of which describe relationships between gene products and molecular and cellular processes. There is, therefore, a growing need to develop automated text-mining techniques to utilize knowledge in the biomedical literature to interpret genome-wide experimental data as well as to aid in the manual curation processes (Rebholz-Schuhmann et al., 2012).

In addition to knowledge extraction, literature mining methods provide a valuable resource for knowledge discovery based on implicit associations in the literature. The concept of literature-based discovery (LBD) was introduced by Swanson several decades ago and is increasingly being discussed in the scientific community (Swanson, 1986; Blagosklonny and Pardee, 2002; Soldatova and Rzhetsky, 2011). Several co-occurrence based-LBD approaches, such as CoPub Mapper (Alako et al., 2005), PubGene (Jenssen et al., 2001), Chilibot (Chen and Sharp, 2004), and GeneWays (Rzhetsky et al., 2004), have been developed. Other approaches have focused on capturing higher order implicit associations, i.e., associations between any pair of entities that do not directly share any abstracts but may share abstracts with other common entities (Burkart et al., 2007). A few approaches have focused on mining TF specific regulatory associations from the literature. Dragon TF association miner (Pan et al., 2004) is a web-based tool that accepts as input a set of abstracts, and identifies and extracts TF associations with Gene Ontology terms found within the text. Natural language processing (NLP) techniques have been used to identify sentences pertaining to transcriptional regulation and to extract relationships from PubMed abstracts for reconstructing regulatory networks (Chen and Sharp, 2004; Šarić et al., 2006; Rodríguez-Penagos et al., 2007; Chen et al., 2014). Vector space models have been investigated in annotation of regulatory networks by prioritizing MEDLINE abstracts likely to have high cis-regulatory content (Aerts et al., 2008). A bootstrapping method has been used to identify gene targets for input TFs (Wang et al., 2011). Additional efforts have concentrated on novel TF discovery by analyzing protein mentions and related contextual information in literature to determine whether a given protein might be a TF (Yang et al., 2009).

Matrix factorization based dimensionality reduction techniques such as singular value decomposition (SVD) and non-negative matrix factorization (NMF) have been used to extract latent functional relationships between genes and terms from the biomedical literature. We previously demonstrated that SVD can extract both explicit (direct) and implicit (indirect) relationships between genes, from the biomedical literature with better accuracy than term co-occurrence methods (Homayouni et al., 2005). Subsequently, we applied this approach to prioritize putative TFs for microarray-derived differentially expressed gene sets (Roy et al., 2011) and to prioritize, cluster, and functionally annotate microRNAs (Roy et al., 2016). The main drawback of SVD is that while it is robust in identifying similarities between entities, it is difficult to determine exactly why they are related. This is due to the fact that the columns of factor matrices can contain negative values, needed to accomplish the best fit numerically in a lower dimensional subspace, which do not have a natural interpretation. As an alternative, non-negative matrix factorization (NMF) was developed to simplify the interpretation of factors by restricting the entries in factor matrices to have non-negative values (Lee and Seung, 1999; Berry et al., 2007). The columns can be interpreted as parts of the original data and the high magnitude entities in the like-numbered column pairs of the two factor matrices can be interpreted as a bicluster. NMF has been used successfully to simultaneously cluster genes along with their related terms (Chagoyen et al., 2006; Heinrich et al., 2008; Tjioe et al., 2010).

Both NMF and SVD can only be applied to two mode data. However, biological networks may contain more than two types of entities whose interactions must be analyzed simultaneously. Tensor factorizations are multiway generalizations of matrix factorizations (De Lathauwer et al., 2000; Kolda and Bader, 2009; Qiao et al., 2017). They have been used in the bioinformatics domain (Luo et al., 2017) to integrate and analyze gene expression data from different sources simultaneously (Omberg et al., 2007; Du et al., 2009; Li and Ngom, 2010; Li et al., 2011; Acar et al., 2012). In the text mining domain, multiway decompositions have been used for clustering chatroom data (Acar et al., 2005), scenario discovery (Bader et al., 2008b), discussion tracking (Bader et al., 2008a), personalized web search (Sun et al., 2005), and web link analysis (Kolda et al., 2005).

Previously, we presented a proof of concept method to simultaneously extract and functionally annotate putative transcriptional modules for a small set of interferon modulated genes (Roy et al., 2014). In this study, we aimed to simultaneously extract genes and their regulatory TFs along with the terms that functionally characterize their relationships on a genome-wide scale. We formulated a 3-mode term × gene × TF tensor containing log-scaled frequencies of terms in abstracts shared between genes and TFs. The tensor was decomposed using non-negative tensor factorization (NTF) into sub-tensors at different low rank approximations. The sub-tensors were interpreted as annotated transcriptional modules (ATMs) consisting of genes and TFs along with the terms that annotate the functional relationship between them. We assessed the validity of the ATMs using GO and KEGG annotations and the performance of the NTF method in literature-based discovery using a set of microarray and ChIP-Seq datasets. We demonstrate that the method can predict downstream target genes for TFs as well as GO classifications based on the knowledge in biomedical literature.

## 2 Materials and Methods

### 2.1 Gene–TF Document Collection

For every mouse gene, PubMed citations were obtained from the gene2pubmed repository available at NCBI. These citations are assigned either by professional staff at the National Library of Medicine or by the scientific research community via Gene Reference into Function (Gene RIF) portal. Since these citations are manually curated, we expect to have a very high precision for tagging correct citations to genes. We further filtered the non-specific citations by removing PMIDs that referred to more than 10 genes as these citations usually described high-throughput experiments mentioning a large number of genes with little to no significant functional information. After filtering, 21,022 mouse genes with at least one assigned citation remained in the collection. Among these, 1,111 genes were identified as TFs in the AnimalTFDB transcription factor database (Zhang et al., 2012). Out of the remaining 19,911 non TF genes, 7,695 genes were found to share at least one citation with at least one TF out of 994 TFs. A total of 45,229 gene–TF pairs with at least one shared citation were identified. For each such gene–TF pair, an abstract document was constructed by concatenation of titles and abstracts for each shared citation. A total of 26,781 unique citations were utilized in creating the abstract documents.

### 2.2 Construction of Term × Gene × TF Tensor

Text to Matrix Generator parser (Zeimpekis and Gallopoulos, 2006) was used to parse terms from the collection of gene–TF documents. All punctuation (excluding hyphens and underscores) and capitalization were ignored. In addition, articles and other common, non-distinguishing words were discarded using a stop list. Terms less than three characters in length were filtered out. A total of 106,895 terms remained after all the filtering. A 3-mode term × gene × TF sparse tensor was created where the entries of the tensor were frequencies of terms in abstracts shared between genes and TFs. Tensor construction from gene–TF documents is depicted in Figure S1 in Supplementary Material using a toy example with a small number of genes, TFs, and terms. The sparse tensor had 5,451,735 non-zero elements out of possible 817,621,682,850 (106,895 × 7,695 × 994) elements resulting in density of 6.66779 × 10^−06^. In order to discount the effect of high frequency common terms in favor of more specific terms that might be better delineators between gene–TF combinations, each tensor entry *f_ijk_* was scaled and transformed into *l_ijk_*:

(1) $$l_{ijk}={\text{log}}_{2}(1+f_{ijk}),$$

where *f_ijk_* is the frequency of the *i*th term in the document corresponding to the *j*th gene and the *k*th TF.

### 2.3 Calculation of Non-Negative Tensor Factorization

Given a 3-mode data tensor *X* of size *m* × *n* × *p*, with *m* mode-1 entities (terms), *n* mode-2 entities (genes), and *p* mode-3 entities (TFs), and a desired approximation rank *k*, the PARAFAC (Harshman, 1970) or CANDECOMP (Carroll and Chang, 1970) model approximates *X* as a sum of *k* rank-1 sub-tensors, each formed by the scaled outer product of a set of three vectors of lengths *m, n*, and *p*. The set of *k* vectors for each mode are usually grouped together in factor matrices *A, B*, and *C* of sizes *m* × *k, n* × *k*, and *p* × *k*, respectively. The columns of the factor matrices are normalized to unit length and the accumulated weight stored in a scaling vector *λ*. In addition, a constraint is imposed on the solution such that *λ*_1_ ≥ *λ*_2_ ≥ … ≥ *λ_k_*. The tensor is expressed as:

(2) $$X=\sum_{i=1}^{k}\;\lambda_{\;i}(a_{i}\circ b_{i}\circ c_{i}),$$

where *a_i_, b_i_*, and *c_i_* represent the *i*th columns of the factor matrices *A, B*, and *C*, respectively; and ◦ denotes the outer product.

A common approach to fitting the PARAFAC model to data is an alternating least squares (ALS) algorithm (Tomasi and Bro, 2006), where one cycles over all factor matrices and performs a least-squares update for one factor matrix while holding all the others constant. We implemented a variant of the PARAFAC model called non-negative tensor factorization (NTF) that constrains the factor matrices to be non-negative. The goal of NTF is to find the best fitting non-negative matrices $A\in {\mathbb{R}}_{+}^{m\times k}$, $B\in {\mathbb{R}}_{+}^{n\times k}$, and $C\in {\mathbb{R}}_{+}^{p\times k}$ in the PARAFAC model that fit the data in *X*, corresponding to the following minimization problem:

(3) $$\underset{A,B,C}{\text{min}}∥X-\sum_{i=1}^{k}\;a_{i}\circ b_{i}\circ c_{i}{∥}_{F}$$

where *F* is the Frobenius norm. The norm of a tensor is similar to that of a matrix:

(4) $$∥X{∥}^{2}\equiv \sum_{i,j,k}\;{\left(x_{ijk}\right)}^{2}.$$

A tensor can be matricized or flattened by rearranging the elements in a matrix. *X*^(^*^m^*^ ×^ *^np^*^)^ represents 1-mode matricization of *X*, which is a matrix of size *m* × *np* where the index *n* runs the fastest over the columns and *p* the slowest. The matricized tensor can be expressed as:

(5) $$X^{m\times np}\approx A(C⊙B{)}^{\prime },$$

where ⊙ represents the Khatri–Rao product (Smilde et al., 2004) and ′ denotes the matrix transpose operator. The Kronecker product of matrices *A* and *B* is given by:

(6) $$A⊗B=\left[\begin{array}{cccc} a_{11}B & a_{12}B & \ldots & a_{1n}B\\ a_{21}B & a_{22}B & \ldots & a_{2n}B\\ \vdots & \vdots & \ddots & \vdots \\ a_{m1}B & a_{m2}B & \ldots & a_{mn}B \end{array}\right]$$

and the Khatri–Rao product is defined as column wise Kronecker product:

(7) $$A⊙B=\left[\begin{array}{cccc} a_{11}b_{1} & a_{12}b_{2} & \ldots & a_{1n}b_{n}\\ a_{21}b_{1} & a_{22}b_{2} & \ldots & a_{2n}b_{n}\\ \vdots & \vdots & \ddots & \vdots \\ a_{m1}b_{1} & a_{m2}b_{2} & \ldots & a_{mn}b_{n} \end{array}\right].$$

The 2-mode and 3-mode matricizations can be similarly expressed as:

(8) $$X^{n\times mp}\approx B(C⊙A{)}^{\prime }$$

(9) $$X^{p\times mn}\approx C(B⊙A{)}^{\prime }.$$

To compute a *k*-factorization with NTF, we first initialized *A, B*, and *C* using the absolute values of *k* leading left singular vectors of the 1-mode, 2-mode, and 3-mode matricizations, respectively. This initialization was performed to make the subsequent NTF computation deterministic as well as to provide a potentially suboptimal starting point that may require less iterations to converge (Boutsidis and Gallopoulos, 2008). Each of the 3 matrices was treated as a non-negative matrix factorization (NMF) sub-problem:

(10) $$\underset{A\in {\mathbb{R}}_{+}^{m\times k}}{\text{min}}∥X^{\left(m\times np\right)}-A(C⊙B{)}^{\prime }{∥}_{F}$$

(11) $$\underset{B\in {\mathbb{R}}_{+}^{n\times k}}{\text{min}}∥X^{\left(n\times mp\right)}-B(C⊙A{)}^{\prime }{∥}_{F}$$

(12) $$\underset{C\in {\mathbb{R}}_{+}^{p\times k}}{\text{min}}∥X^{\left(p\times mn\right)}-C(B⊙A{)}^{\prime }{∥}_{F}$$

and solved in succession using the multiplicative update rule (Welling and Weber, 2001) modified to incorporate ∈ for stability. For example, to solve for *A* the update rule is:

(13) $$A_{i\rho }\leftarrow A_{i\rho }\frac{{\left(X^{\left(m\times np\right)}Z\right)}_{i\rho }}{{\left(AZ^{\prime }Z\right)}_{i\rho }+\in },\;Z=(C⊙B).$$

**Figure 1:**
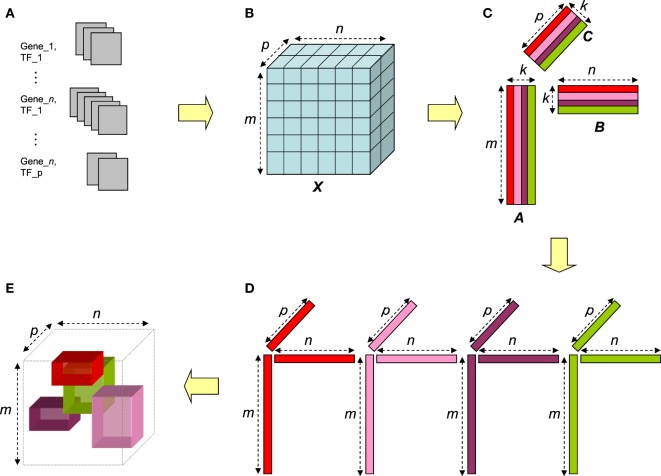
Overview of the NTF-based procedure. Gene–TF documents **(A)** are parsed to construct term × gene × TF tensor **(B)**. The tensor is factorized via NTF to generate non-negative factor matrices for terms, genes, and TFs **(C)**. For a given approximation rank *k*, the entities corresponding to the high magnitude entries in each triad of columns (one from each matrix) **(D)** can be interpreted as an annotated transcriptional module (ATM) comprising of genes and TFs functionally annotated by the corresponding terms **(E)**.

The ∈ is a small number 10^−9^ added to the denominator in order to add stability to the calculation and guard against introducing a negative number from numerical underflow. The approximation rank *k* > 0 of NTF corresponds to the number of 3-way associations (sub-tensors) whose additive contributions approximate the information in the data tensor. Each triad {*a_i_, b_i_, c_i_*}, for *i* = 1, …, *k*, defines scores for a set of terms, genes, and TFs for a particular 3-way association in the corpus. The scaling factor *λ_i_* (after normalization) indicates the weight of the association for triad *i*. NTF was applied to the term × gene × TF log weighted frequency tensor using functions from MATAB tensor toolbox (Bader and Kolda, 2012). The procedure is described graphically in Figure 1.

### 2.4 Interpretation of Sub-Tensors As Annotated Transcriptional Modules (ATMs)

A *k*-factorization delivers *k* sub-tensors. A sub-tensor *i* can be reconstructed via the outer products of the *i*th columns of the three non-negative factor matrices corresponding to terms, genes and TFs, respectively. For each such triad, the entities corresponding to the high magnitude scores in each column contribute more to the information content in the sub-tensor than the low magnitude ones and are, therefore, deemed more significant. We construed the triplet of sets of such significant genes, TFs, and terms as an annotated transcriptional module (ATM). Each ATM contained genes and TFs representing dominant elements of a putative transcriptional network, along with terms describing the functional interaction between these genes and TFs.

In order for the ATMs to be consistent with observed biological networks in terms of the number of entities, we established upper bounds for the number of genes, TFs, and terms in the ATMs. We observed the distribution of numbers of genes and TFs in 249 KEGG pathways and found a maximum of 184 genes and 21 TFs in a single pathway (after excluding the outliers). For the terms, we chose the upper bound of 300 as this is usually the maximum number of words allowed for publication abstracts and may be adequate to describe a transcriptional network.

For a given column and an upper bound *n*, we first obtained a truncated list *D* containing *n* highest scoring entities. We then calculated the number of significant (high magnitude) entities required to approximate the information content in the list as described in Alter et al. (2000). Briefly, contributions of each score *g_i_* were computed as $p_{i}=\frac{g_{i}}{S}$, where $S=\sum_{j=1}^{n}\;g_{j}$ (sum of all scores). Subsequently, we calculated the normalized entropy of the list as:

(14) $$E=\frac{-\sum_{i=1}^{n}\;p_{i}log(p_{i})}{log(n)},$$

which is the fraction of information content in the list relative to a completely random list of the same size. The number of significant entities *s* was calculated as:

(15) $$s=max\left(1,\left|p_{i}>\frac{1}{n\cdot E}\right|\right).$$

### 2.5 Performance Evaluation of ATMs

Functional enrichment analysis was performed using two different human curated datasets. For the first set, we downloaded the list of 249 mouse related manually curated pathways and their associated genes present in the Kyoto Encyclopedia of Genes and Genomes (KEGG) (Kanehisa et al., 2004). For the second set, we downloaded the list of 12,418 mouse related Gene Ontology (GO) (Ashburner et al., 2000) categories and their associated genes. The set of genes and TFs belonging to each ATM was evaluated for functional enrichment in the aforementioned KEGG pathways and GO categories using a hypergeometric test as described in Tavazoie et al. (1999). As a control, we compared the enrichment frequencies of ATMs to a set of 1,000 randomly generated gene–TF sets containing 8 genes and 2 TFs from the tensor. The numbers 8 and 2 depict the median number of genes and TFs across all 2,861 ATMs. To create each random gene–TF set, 8 genes out of 7,695 genes constituting the tensor were randomly picked. Similarly 2 TFs were randomly picked from 994 TFs in the tensor. This process was repeated 1,000 times.

The area under the curve (AUC) was used as a measure of quality of the terms associated with ATMs. The AUC will have the value of 1 for perfect ranking (all relevant terms at the top), 0.5 for randomly generated ranking, and 0 for the worst possible ranking (all relevant terms at the bottom) (Hanley and McNeal, 1982). The terms in the descriptions of KEGG and GO categories served as the gold standards.

Precision was used as a measure of accuracy of the gene–TF associations in an ATM. It was calculated as the ratio of the number of common entities (genes and TFs) between an ATM and the gold standard set, to the number of entities in the ATM. Mathematically, the formula was defined as:

(16) $$\frac{\left|\{\mathrm{genes and TFs in ATM}\}\cap \{\mathrm{genes and TFs in gold standard set}\}\right|}{\left|\{\mathrm{genes and TFs in ATM}\}\right|}.$$

In addition, a set of ATM precision values was tested for significance by computing a right-tailed two-sample Welch’s *t*-test (Press, 1992) between the precisions for the ATMs being evaluated, and the precisions for 200 × *n* randomly generated gene–TF sets from the tensor. For each of *n* ATMs being evaluated, 200 gene–TF sets were randomly generated containing the same distribution of genes and TFs as the ATM.

Redundancy between any two sets of entities of the ATMs was computed as the Jaccard coefficient between the sets, which is defined as the ratio of the number of elements in the intersection and the number of elements in the union. The Jaccard coefficient will have a value of 0 for disjoint sets and a value of 1 for duplicate sets.

## 3 Results

Unlike other matrix factorization approaches, it is computationally difficult to estimate the true rank of a tensor (Håstad, 1990). Therefore, we computed NTF at 16 approximation ranks *k* = 1, 2, 3, 5, 10, 15, 20, 25, 30, 50, 100, 200, 300, 500, 700, and 900. For every *k*, NTF required less than 60 iterations to satisfy a tolerance of 10^−4^ in the relative change of fit. For a given approximation rank *k*, all genes, TFs, and terms in each of the *k* sub-tensors were ranked based on their scores as demonstrated in Figure 1. We then used an entropy based method to determine the score threshold and to define relevant genes, TFs, and terms within an annotated transcriptional module (ATM) as described in Materials and Methods. Figure S2 in Supplementary Material shows the frequency distribution of genes, TFs, and terms computed for each of the 2,861 ATMs produced across all *k*s. The median number of TFs (~2) and terms (~60) was stable across all *k* factorizations. In contrast, the median number of genes in ATMs decreased with increasing *k*. A total of 3,608 unique genes, 772 unique TFs, and 11,697 unique terms occurred across all 2,861 ATMs.

### 3.1 Tensor Landscape As a Function of Approximation Rank *k*

A *k*-factorization expresses the three-way gene regulatory interaction information in the tensor as additive superposition of latent information content in *k* sub-tensors. An approximation with *k* = 1 is expected to summarize the information content as the most dominant (or representative) transcriptional network gleaned from the literature, while *k* = 2 expresses the information content as superposition of two most dominant transcriptional networks. As *k* increases, the factorizations are expected to reveal more specific networks.

**Figure 2:**
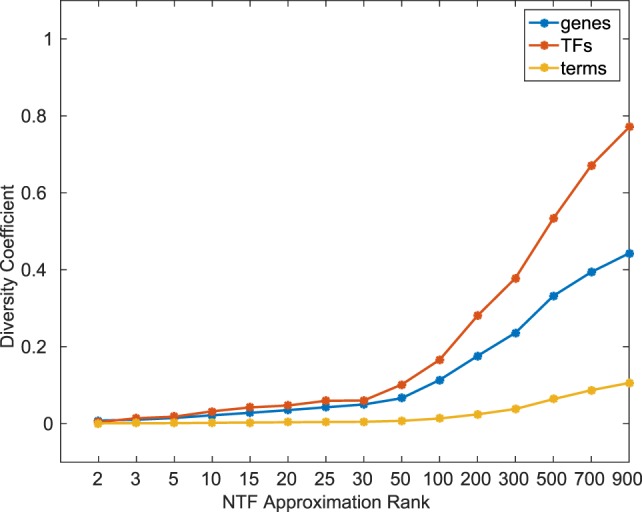
Diversity coefficients of genes, TFs, and terms in ATMs across various *k*-factorizations.

In order to quantitatively examine the information content across different *k*, we calculated a diversity coefficient *d* for each type of entity in the ATMs obtained at various *k* factorizations. For each entity type, we defined the diversity coefficient as the ratio of the number of unique entities in the union of all *k* ≥ 2 ATMs, and the total number of entities in the tensor. Figure 2 shows the diversity coefficients for genes, TFs, and terms. As expected, we found that the diversity of TFs, genes, and terms in the ATMs increased with increasing *k*. In addition, we computed the pairwise redundancy using the mean Jaccard coefficient between any two sets of entities in the ATMs for *k* ≥ 2 (Figure S3 in Supplementary Material). We found that while the overall diversity increases with increasing *k*, the pairwise redundancy between the entities remained constant, and that for any given *k*-factorization, the ATMs were disjoint. Taken together, these results indicate that at higher *k*, we were able to extract unique regulatory modules with more specific functional annotations.

### 3.2 Functional Validation of ATMs

**Figure 3:**
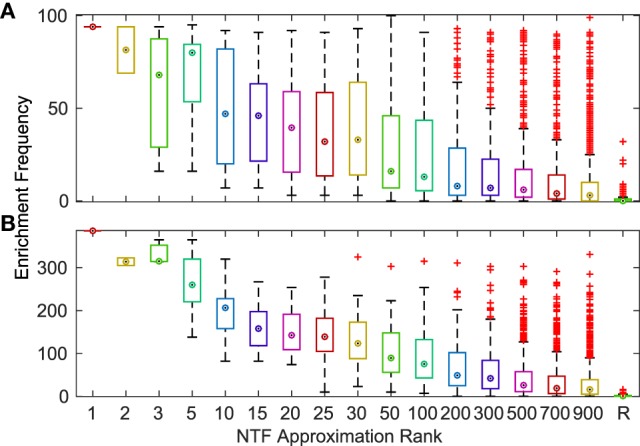
Frequency of significantly (*p* ≤ 0.05) enriched KEGG **(A)** and GO **(B)** categories for ATMs across various *k*-factorizations. As a control, the enrichment frequency of 1,000 randomly (R) generated gene sets are shown at the far right of each panel.

To comprehensively evaluate the functional relevance of ATMs generated by our method, we examined if the genes and TFs in the ATM were significantly (p-value ≤ 0.05, hypergeometric test as described in Materials and Methods) enriched in KEGG or GO categories. We found that more than 94% of the 2,861 ATMs were enriched in at least one KEGG or GO category. This result indicates that tensor factorization across all *k*s produced biologically relevant ATMs. The median number of enriched categories in all ATMs at each *k* value was greater than chance (Figure 3). Also, the number of enriched KEGG and GO categories per ATM was higher at lower *k*. This result indicates that at lower *k*, ATM genes, and TFs have a broad range of functions, consistent with our earlier observation that the diversity and specificity of ATMs increased with higher *k* (Figure 2; Figure S3 in Supplementary Material). Consistent with these observations, we found that the diversity of enriched KEGG and GO categories in ATMs increased at higher *k* (Figure S4 in Supplementary Material), while the pairwise redundancy between categories remained low as *k* increased (Figure S5 in Supplementary Material). Taken together, these results suggest that the gene–TF ATMs are more functionally specific at higher *k*.

Interestingly, we found a higher diversity of KEGG pathways than GO categories at all *k* values (Figure S4 in Supplementary Material). This result suggests that KEGG pathways can be more specific than GO categories. Some KEGG and GO categories were very frequent among all ATMs. For instance, among the top ten overrepresented KEGG pathways, five were related to cancer, four related to infection, and one related to cytokine signaling (Table S1 in Supplementary Material). On the other hand, among the top ten overrepresented GO categories, four were related to development, three were related to regulation of gene expression, two related to cell proliferation, and one related to protein phosphorylation (Table S2 in Supplementary Material).

**Figure 4:**
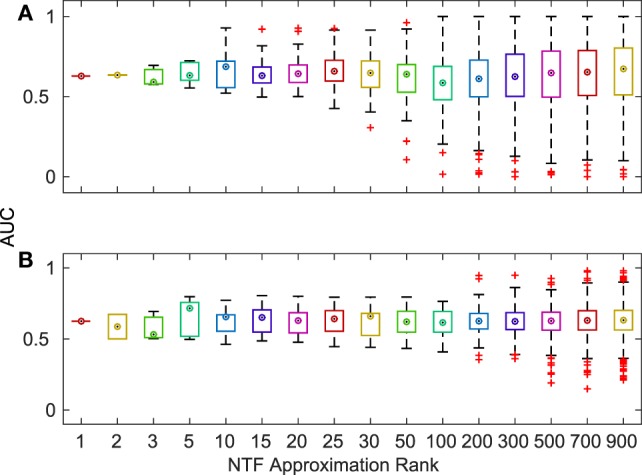
Distribution of AUCs for the terms associated with each ATM across various *k*-factorizations against KEGG **(A)** and GO **(B)** category titles and descriptions.

Finally, we evaluated the accuracy of the terms identified by NTF for each ATM by comparing them with KEGG and GO annotations. For each ATM enriched in at least one KEGG pathway or GO category, we created a gold standard using all of the terms in the titles and descriptions of the enriched pathways and categories. We compared the top ATM terms against these gold standards by examining the area under the curve (AUC) of receiver operating characteristics (ROC) curves. We found that more than 80% of the 2,861 ATMs produced AUCs above 0.6. There was no change in median AUC across *k*, albeit the range of AUCs increased with increasing *k* (Figure 4). The deviation of the median AUC from the lowest point increased faster with increasing *k* than the deviation from the highest point. This indicates that at higher *k*, some NTF-derived functional annotations are much less consistent with the descriptors of KEGG and GO categories.

### 3.3 Prediction of Gene–TF Associations

A unique advantage of our approach is that it enables literature based discovery, i.e., new potential gene–TF interactions may be deduced from implied associations in the literature in the absence of direct (experimental) evidence. To evaluate the performance of our method in predicting potentially new gene–TF relationships, we used two different types of experimental data as gold standards. Chromatin immunoprecipitation sequencing (ChIP-Seq) is a genome wide technology that identifies direct binding sites for specific transcription factors on gene promoters. On the other hand, microarray expression analysis of tissues with a targeted deletion of specific transcription factor identifies downstream genes whose expression levels are either directly or indirectly altered by the transcription factor.

We used a set of previously published ChIP-Seq data for four transcription factors (*Cebpα, E2f4, Foxa1*, and *Foxa2*) in either liver or 3T3-L1 cells (MacIsaac et al., 2010) as gold standard validation sets. To evaluate our method, we focused only on the ATMs which contained the gold-standard TF. The number of ATMs for the four gold-standard TFs ranged from 11 to 54 and the number of genes and TFs in all the ATMs for the gold-standard TFs ranged from 34 to 112 and 8 to 45, respectively (Table 1). To calculate the average precision for each gold-standard TF, we compared the union of genes and TFs in all ATMs against the ChIP-seq validation set. The number of genes in the four validation sets ranged from 938 to 8,595 genes. As shown in Table 1, the average precision of our method ranged between 11% (*Foxa1*, liver) and 91% (*E2f4*, 3T3-L1). Except for *Foxa2*, all gene–TF associations extracted by our method were significantly (*p* ≤ 0.05, Welch’s *t*-test) higher than chance. The average precisions for a given TF in two different tissues varied, indicating that binding sites are tissue dependent. These results indicate that NTF is accurate for identifying TF target genes from the biomedical literature. Importantly, we found that 14–39% of the target genes predicted by our method were based on implied associations in the literature. An explicit association refers to the cases when a gene and a TF share an abstract, whereas an implicit association is inferred based on shared terms among genes or TFs in the same ATM. This result suggests that our method is useful for making literature based discoveries.

**Table 1:** Performance of NTF using ChIP-Seq data sets.

TF (GEO accession ID)	Tissue	ATM count	Genes in all ATMs	TFs in all ATMs	Genes in validation set	Average precision	p-Value	Explicit%	Implicit%
Cebpa (GSM427088)	Liver	13	48	11	8,411	0.49	7.26E−05	0.86	0.14
Cebpa (GSM427093)	3T3-L1 cells	13	48	11	2,836	0.34	9.39E−06	0.82	0.18
E2f4 (GSM427091)	Liver	22	34	8	7,134	0.82	5.08E−16	0.69	0.31
E2f4 (GSM427094)	3T3-L1 cells	22	34	8	8,595	0.91	4.71E−17	0.61	0.39
Foxa1 (GSM427090)	Liver	11	47	9	938	0.11	3.46E−02	0.71	0.29
Foxa2 (GSM427089)	Liver	54	112	45	6,366	0.21	9.14E−01	0.74	0.26

Our literature mining method identifies gene–TF associations based on functional information in the biomedical abstracts. Thus, it is possible that some of the gene predictions by our method are not the primary gene targets of the TFs, but rather genes in similar functional pathways whose expression levels are affected indirectly via a downstream TF. To test this possibility, we evaluated our method using four microarray datasets as gold standards. These datasets include differentially expressed genes in different mouse tissues (cerebellum, retina, or choroid plexus) as a consequence of targeted deletions in specific TFs (*Atoh1* (Ha et al., 2015), *Pax6* (Ha et al., 2015), and *Otx2* (Omori et al., 2011; Johansson et al., 2013)). The number of genes and TFs in all the ATMs for the gold-standard TFs ranged from 57 to 152 and 34 to 62, respectively (Table 2). The number of genes in the validation sets ranged from 2,137 (*Atoh1*, cerebellum) to 11,689 (*Otx2*, choroid plexus). The average precision for all four validation sets ranged from 0.33 to 0.42 and were all significantly (*p* ≤ 0.05, Welch’s *t*-test) higher than chance. Importantly, approximately 27–63% of the predictions from our method were based on implied associations extracted from the literature. Since transcriptional regulation by TFs are tissue specific, we narrowed the ATMs to those which explicitly mentioned the experimental tissue. We calculated average precision for ATMs that contained either one or two keywords associated with the tissue. In all but one case (*Otx2*, choroid), the average precision values improved when we considered only the tissue-relevant ATMs.

**Table 2:** Performance of NTF using microarray data sets.

TF knockout (GEO ID)	Tissue	# of ATMs	Total # genes	Total # TFs	# Genes in validation set	Average precision			Base ATM associations (%)	
						Base (p-value)	Base + (keyword)	Base + (keywords)	Explicit	Implicit
Atoh1	Cerebellum	38	73	36	2,137	0.33 (*p* < 9.07*E*−07)	0.50 (cerebellum)	0.71 (cerebellum, rhombic)	0.37	0.63
Pax6	Cerebellum	74	152	62	3,036	0.38 (*p* < 2.87*E*−18)	0.54 (cerebellum)	0.57 (cerebellum, rhombic)	0.73	0.27
Otx2 (GSE21900)	Retina	41	57	34	6,181	0.36 (*p* < 5.31*E*−06)	0.523 (retina)	–	0.51	0.49
Otx2 (GSE27630)	Choroid plexus	41	57	34	11,689	0.42 (*p* < 3.1*E*−05)	0.28 (choroid)	–	0.68	0.32

*Average precision values were determined for all ATMs which included the target TF (base) or base modules that also included one or two keywords associated with the experimental tissue. An explicit association refers to the cases when a gene and a TF share an abstract, whereas an implicit association is inferred among genes or TFs in the same ATM*.

**Figure 5:**
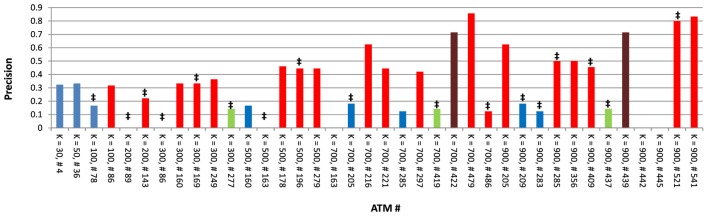
Precision values for all 38 *Atoh1* ATMs. ^‡^ denotes ATMs in which *Atoh1* was ranked first; red bars denote ATMs that include the term “cerebellum”; green bars denote ATMs that include the term “rhombic”; brown bars denote ATMs which include both “cerebellum” and “rhombic” as terms; and blue bars denote ATMs that include neither “cerebellum” or “rhombic” as terms.

Next, we focused on one dataset (*Atoh1*) to carefully examine the characteristics of each ATM. As indicated above, the average precision across all 38 *Atoh1* ATMs was 0.33. However, the precision of individual *Atoh1* ATMs ranged between 0 and 0.85 (Figure 5). In general, higher *k* produced higher precision, indicating that higher classification specificity is correlated with precision. Interestingly, precision was not correlated with whether *Atoh1* was ranked first in the ATM. The average precision was 0.23 for the 17 ATMs in which *Atoh1* was top ranked. In contrast, *Atoh1* was first ranked in only one of the top three ATMs with the highest precision (Figure 5). Finally, six ATMs had a precision of 0. Upon examination, we found that these ATMs were significantly enirched (*p* ≤ 0.05) for “auditory receptor cell differentiation” and other GO categories related to ear development. Consistent with these GO classifications, the terms in the ATMs were related to hair cell differentiation and development. Thus, it is likely that the precision of these ATMs is low because the gold standard was derived from microarray experiments using cerebellar tissue rather than auditory tissue.

Finally, as a benchmark, we compared the predictions of our method with those obtained by RegNetwork (Liu et al., 2015) for the same experimental ChIP-Seq and TF knockout microarray datasets described above as gold standards. RegNetwork is a database containing target predictions for TFs, which have been sourced from more than 20 interaction databases. Tables S3 and S4 in Supplementary Material show the precisions obtained by NTF along with those obtained by RegNetwork predictions. With the ChIP-Seq gold standards, we found that the average precision of NTF method was 0.48 compared to 0.38 for RegNetwork. NTF outperformed in 4 out of 6 ChIP-Seq datasets. For the two other datasets, RegNetwork outperforms but only marginally. In contrast, with the TF knockout microarray gold standards, the average precision of NTF (0.37) was better than that of RegNetwork (0.16). NTF precision was higher for 3 out of the 4 microarray datasets. Importantly, RegNetwork predicted targets for *Foxa1* and *Otx2* did not match any targets in the gold-standard datasets, whereas NTF identified target genes with 0.11 and 0.42 precision, respectively (Tables S3 and S4 in Supplementary Material).

### 3.4 Prediction of Functional Classifications

Current methods for functional interpretation of high-throughput genomic data rely on manually curated knowledge bases, such as GO, KEGG, and a variety of other resources. It is generally accepted that the rate of manual curation is not sufficient for the amount of genomic data that is being generated in various species (Baumgartner et al., 2007). Moreover, there is a quantifiable bias in manually curated knowledge bases toward more popular genes and a substantial drift in the annotation of genes over time (Gillis and Pavlidis, 2013). Previous work have focused on using text-mining approaches to enhance curation of GO and other knowledge bases (Chagoyen et al., 2006; Couto et al., 2006; Thomas et al., 2015; Peng et al., 2016).

Here, we examine the performance of our NTF approach in predicting (suggesting) GO classifications. GO predictions were based on two approaches: (1) Guilt-by-Association (GBA) or (2) term mapping. In the GBA approach, new genes or TFs were assigned to a GO category which is significantly enriched for one or more ATMs. These new genes or TFs are part of the ATMs but have not been explicitly assigned to the enriched GO category by the curators. In the term mapping approach, novel and more specific GO categories for a given ATM were predicted based on the number of terms from the ATM that overlapped with GO category descriptions. These new GO categories were not significantly enriched for the ATM under consideration. The results for both approaches were evaluated by manual examination of the biomedical literature.

In order to evaluate the GBA approach, for the 38 ATMs containing *Atoh1*, we randomly chose 3 GO categories that were found to be significantly enriched in some of those ATMs (Table 3). For example, for category “Axon guidance” that was significantly enriched for 24 ATMs containing a total of 104 genes and TFs, only 16 genes and/or TFs were explicitly assigned to the category by the GO curators. Upon manual examination of sentences in Medline abstracts, we found that out of the 104 genes and TFs, 29 were functionally related to axon guidance. Among the 29, 11 were already assigned to the GO category but the rest (18) were not. The latter are candidates for assignment to the “axon guidance” category, potentially increasing the assignment of new genes and TFs to the category by 1.6-fold. Five of the existing GO assignments could not be validated through manual evaluation. Overall, for three randomly selected significantly enriched GO categories (Table 3), the GBA method increased the assignment of new genes by an average of 1.2-fold. The average precision for the GO assignment by this method was 0.27 (0.14–0.38 range). Notably, on average, 52% (26–100% range) of the existing GO annotations were not validated by our manual analysis, indicating that there is substantial error in GO curation (Table 3).

**Table 3:** Significantly enriched GO categories for *Atoh1* modules for evaluating guilt-by-association (module membership).

GO category (# of enriched ATMs)	ATM genes #	GO curated		Manual validation	
		Assigned	Unassigned	Assigned	Unassigned
Axon guidance (24)	104	16	88	11	18
Neuron migration (15)	78	15	63	11	19
Neural crest cell migration (1)	35	4	31	0	5

In order to evaluate the term mapping approach, for the 38 ATMs containing *Atoh1*, we predicted 7 novel GO categories whose names and descriptions were found to have high overlap with the terms from some of those ATMs (Table 4) but were not significantly enriched for any ATM. For example, for category “Spinal cord oligodendrocyte cell differentiation” whose description had high overlap with the terms of 3 ATMs containing a total of 44 genes and TFs, only 1 gene or TF was explicitly assigned to the category by the GO curators. This low number contributes to the reason the category was not significantly enriched for any of the 3 ATMs. Upon manual examination of sentences in Medline abstracts, we found that out of the 44 genes and TFs, 9 were functionally related to spinal cord oligodendrocyte cell differentiation. Among these, 1 was already assigned to the GO category but the rest (8) were not. The latter genes and TFs are candidates for assignment to the category, potentially increasing the assignment of new genes and TFs to the category by 8-fold, which make the category a plausible candidate for being significantly enriched for the ATMs whose terms have high overlap with the category description. On average, 3.9-fold (1- to 8-fold range) more genes were assigned to novel predicted GO categories, but with an average precision of 0.15 (0.06–0.23 range) (Table 4). Most of these categories were more specific than the currently enriched categories.

**Table 4:** Predicted GO categories for *Atoh1* modules based on term mapping.

GO category (# of ATMs with high term overlap)	ATM genes #	GO curated		Manual validation	
		Assigned	Unassigned	Assigned	Unassigned
Spinal cord oligodendrocyte cell differentiation (3)	44	1	43	1	8
Central nervous system vasculogenesis (1)	35	1	34	0	2
Cajal–Retzius cell differentiation (2)	24	0	24	–	2
Roof plate formation (3)	13	1	12	1	2
Schwann cell development (3)	15	0	15	–	2
Cerebellar granular layer development (6)	34	0	34	–	7
Cerebellar Purkinje cell layer development (7)	34	0	34	–	5

**Figure 6:**
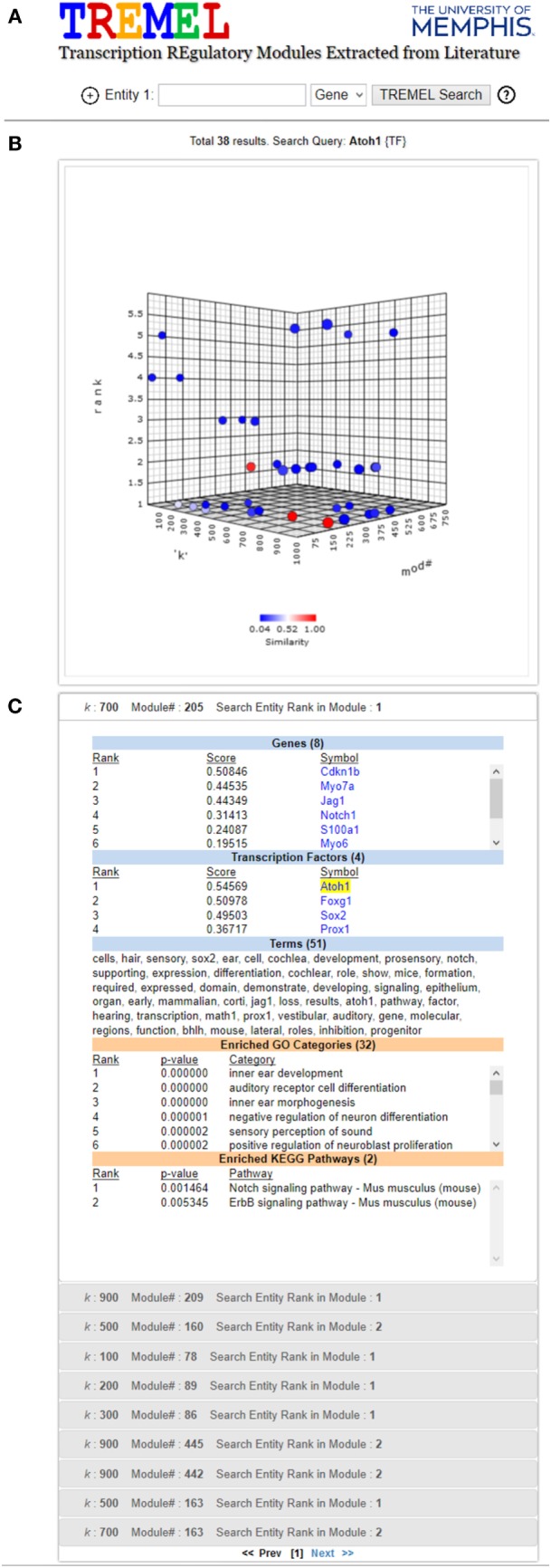
Screen shot of TREMEL (Transcription REgulatory Modules Extracted from Literature) tool. **(A)** Search feature allows the user to query genes, TFs, or terms across all 2,861 ATMs. In addition, complex queries involving a combination of any of the three entities can be performed by adding another query box. **(B)** Overview display shows all of the ATMs which relate to the query with respect to the rank of the query entity, *k*, and ATM number. **(C)** Displays the ranked genes, TFs, and terms for the selected ATM in panel **(B)**. In addition, the enriched GO and KEGG categories are displayed in order for the user to quickly compare the NTF terms against human curation.

In general, the terms associated with ATMs provide a greater level of functional specificity than structured categories in ontologies. For example, Figure 6C shows the genes, TFs, and terms associated with one ATM in which *Atoh1* is top ranked. As a comparison, the enriched GO and KEGG categories are also displayed. Whereas the GO categories indicate that this group of genes and TFs are associated with inner ear development and auditory receptor cell differentiation, the terms (shown in *italics* below) in the ATM suggest that they are involved in *differentiation* of the *sensory hair cells* in the *cochlea* located in the *organ* of *Corti*. *Atoh1* is a basic helix loop helix (*bhlh*) *domain transcription factor* involved in *early development*. Targeted deletion of *Atoh1* gene in *mice results* in *loss* of *hair cells* in the *organ* of *Corti* (Chonko et al., 2013). Several gene symbols also appear in the top ranked terms such as *Sox2, Notch, Jag1, Prox1*, and *Math1*. Interestingly, *Math1* is the alias for *Atoh1*. Direct evidence suggests that *Notch1* and *Jag1 signaling pathway* is required for activation of *Sox2* and *Atoh1 expression* (Neves et al., 2011). Activation of *Atoh1* by *Sox2 transcription factor* is required for *hair cell development* in *cochlea* with respect to both expansion of the *progenitor cells* in the *cochlear epithelium* and initation of *hair cell differentiation* (Kiernan et al., 2005; Kempfle et al., 2016). Conversely, *Prox1 transcription factor* directly suppresses *Atoh1 expression*. *Sox2* is *expressed* in type 2 *vestibular hair cells* and in *supporting cochlear* and *vestibular epithelium* (Hume et al., 2007).

### 3.5 TREMEL Web Tool

In order to facilitate manual examination of individual ATMs, e.g., the aforementioned *Atoh1* ATMs, the publicly available web tool called Transcriptional Regulatory Modules Extracted from Literature (TREMEL) was developed. It is accessible at http://binf1.memphis.edu/tremel. TREMEL provides a searchable interface for the genes, TFs, and terms for all 2,861 ATMs (Figure 6).

The user can query the tool with either genes, TFs or terms, or a combination of any of the 3 types of entities in additional query boxes. The entity type can be selected from the drop down list to the right of each search box. Clicking the “+” button to the left of the first search box opens an additional search box. A maximum of 3 search boxes are allowed. The gene and TF queries need to be the official symbols designated by the National Center for Biotechnology Information (NCBI). Only one symbol is allowed per search box. The term query can be any single keyword such as “cancer,” “neuron,” “transcription,” etc. All queries are case insensitive. The output of the tool consists of two panels.

The top panel is comprised of a 3-dimensional interactive plot that shows all ATMs containing the search box entities, as points. The axes of the plot correspond to the NTF approximation rank *k*, the ATM#, and the rank of the queried entity (first search box only) in the ATMs. An ATM can be selected in the panel by clicking on its corresponding point in the 3-D plot. The color of the selected point changes to red and the colors of the remaining points corresponding to all other ATMs are depicted in terms of similarity to the selected ATM. The most similar ATMs are colored in shades of red while the least similar ones are colored in shades of blue. The similarity between any two ATMs is calculated as the Jaccard coefficient between the sets of genes and TFs in the respective ATMs. Upon initial search completion, one ATM is preselected and colored in red. This initial selection is performed in a manner such that the ATM with the lowest queried entity rank is picked. Ties are resolved in favor of the ATM with the lowest NTF approximation rank.

The bottom panel contains several sub-panels, each corresponding to an ATM point in the first panel. The top sub-panel corresponds to the selected ATM, and the remaining ATMs are ordered according to their similarity to the selected ATM. Each sub-panel displays the ranked genes, TFs and terms of the corresponding ATM, as well as the enriched GO categories and KEGG pathways. Clicking a sub-panel expands it to display its contents, and closes the previously open sub-panel. The contents of only one sub-panel are viewable at a time.

## 4 Discussion

We have shown for the first time that NTF can be used effectively to simultaneously extract and functionally annotate transcriptional networks from the biomedical literature on a genomic scale. NTF is able to generalize and overcome data sparsity to produce interpretable low rank approximations. The ATMs comprised of genes, TFs, and terms were evaluated by several approaches. More than 94% of the gene–TF ATMs were enriched in at least one KEGG or GO category. In addition, there were considerable overlap (AUC values above 0.6) between the NTF-derived terms and the descriptions of the enriched KEGG and GO categories. Importantly, NTF identified more specific terms related to genes and TFs than what is currently available in KEGG and GO databases (Table 4; Figure 6). Thus, our approach provides more flexibility for scientists to search with a broader range of terms to identify relevant transcriptional networks directly from the literature. To assist researchers in exploring and discovering functional insights about transcriptional networks, we developed the web tool TREMEL, which provides a searchable interface for the genes, TFs, and terms for all 2,861 ATMs (Figure 6).

We validated our NTF derived ATMs using a number of different data sources, including GO (manually curated), KEGG (manually curated), ChIP-Seq (experimental), and TF knockout microarray (experimental). We also compared our gene–TF association predictions with those obtained by RegNetwork and found that overall, the NTF method produced comparable or better precision (specificity) compared to the RegNetwork. However, calculating recall (sensitivity) using these data sources may not be appropriate because none of the data sources are 100% accurate and complete. Manual curation is known to be incomplete (Baumgartner et al., 2007) and high-throughput experimental datasets are highly variable for technical reasons. Indeed, in Table 4 we show a few examples where NTF term annotations were more specific and relevant than the enriched GO categories. In addition, we showed that NTF performed comparably to or better than human curated RegNetwork, which is an aggregated database of TF–gene interactions. However, unlike the NTF approach, predicted targets of RegNetwork for two out of ten transcription factors examined did not match any targets in the gold-standard datasets (Tables S3 and S4 in Supplementary Material). Finally, since the NTF literature mining approach extracts conceptual and functional associations between TFs and genes, we would not expect very high precision with any of the experimental benchmarks for several reasons. Genomic assays are highly tissue specific, whereas the functional associations in the literature may not necessarily be focused on a specific tissue. ChIP-seq assays detect only direct interaction between TFs with gene promoters, whereas the microarray experiments identify both direct and indirect TF–gene associations. Finally, both ChIP-Seq and microarray experiments are prone to false-discovery due to multiple hypothesis testing.

As estimation of the true tensor rank is computationally difficult, we opted for a more exploratory approach where we evaluated factorization at several approximation ranks (*k* ranging from 1 to 900). KEGG/GO pathway divergence analysis (Figure S3 in Supplementary Material), and experimental data validation (Figure 5) and manual evaluation indicated that more specific pathways are delineated at higher *k*. Consistent with this notion, we found that the median number of enriched KEGG/GO categories were substantially lower at higher *k* (Figure 3). On the other hand, this seemingly low number of enrichment could be partially due to shortcomings of KEGG and GO annotations as we demonstrated after manual analysis (Tables 3 and 4) and previously documented by Gillis and Pavlidis (2013).

The interpretation of sub-tensors in our method allows for redundancy in ATMs, where certain entities may occur in multiple ATMs from the same *k*-factorization. We believe that this makes biological sense as a TF might be involved in different functions or pathways depending on the set of genes with which it interacts. For instance, analysis of *Atoh1* ATMs revealed that some of its ATMs were related to early cerebellar development, whereas other ATMs were related to hair cell development in the auditory system. This approach is an improvement over previous work by our group and others using NMF (Heinrich et al., 2008; Tjioe et al., 2010), where the factors were interpreted to have only disjoint biclusters and a set of genes were associated with the best set of associated terms.

Taken together, our results demonstrate that NTF is a promising technique to simultaneously extract genes, TFs and related terms to identify and predict gene regulatory networks from the biomedical literature. The method and tool presented here are not intended to be comprehensive (high recall) nor a replacement of high-throughput experiments. However, we have shown that it is a valuable exploratory tool which can help researchers make new mechanistic discoveries and help human curators to quickly annotate gene function based on the vast amount of knowledge in the biomedical literature. In addition, our work sets the stage to apply this technique to other areas in systems biology such as simultaneous extraction of terms with miRNAs and their target genes, small molecules and protein targets, as well as drugs and diseases.

## Author Contributions

SR and RH designed the research and wrote the manuscript. SR and BM analyzed the results. DY developed the web tool. MB and L-YD contributed mathematical and statistical methods. DG contributed biological experimental data for validation. RH supervised the research and assisted with the interpretation of results. All authors reviewed the manuscript.

## Conflict of Interest Statement

The authors declare that the research was conducted in the absence of any commercial or financial relationships that could be construed as a potential conflict of interest.
