# Supplementary material for: Navigating the Functional Landscape of Transcription Factors via Non-Negative Tensor Factorization Analysis of MEDLINE Abstracts
Source: Front Bioeng Biotechnol. 2017 Aug 28;5:48. doi: 10.3389/fbioe.2017.00048 (PMC5581332; doi:10.3389/fbioe.2017.00048)
Supplement: Supplementary file 1 [file Data_Sheet_1.PDF]

Supplementary Material: Navigating the Functional Landscape of Transcription Factors via Non-Negative Tensor Factorization Analysis of MEDLINE Abstracts

1 SUPPLEMENTARY TABLES AND FIGURES

1.1 Supplementary Tables

Table S1. 10 KEGG pathways that were enriched most in ATMs across k.

KEGG ID	Description	Frequency
5200	Pathways in cancer - Mus musculus (mouse)	1309
5166	HTLV-I infection - Mus musculus (mouse)	830
5215	Prostate cancer - Mus musculus (mouse)	707
5169	Epstein-Barr virus infection - Mus musculus (mouse)	682
4010	MAPK signaling pathway - Mus musculus (mouse)	651
5152	Tuberculosis - Mus musculus (mouse)	651
4060	Cytokine-cytokine receptor interaction - Mus musculus (mouse)	637
5220	Chronic myeloid leukemia - Mus musculus (mouse)	623
5202	Transcriptional misregulation in cancer - Mus musculus (mouse)	602
5218	Melanoma - Mus musculus (mouse)	600

Table S2. 10 GO categories that were enriched most in ATMs across k.

GO ID	Description	Frequency
10628	positive regulation of gene expression	870
42127	regulation of cell proliferation	699
7507	heart development	691
9887	organ morphogenesis	691
51091	positive regulation of sequence-specific DNA binding transcription factor activity	633
8083	growth factor activity	611
30324	lung development	586
8283	cell proliferation	569
1934	positive regulation of protein phosphorylation	568
10468	regulation of gene expression	546

Table S3. Comparison of precision values between NTF and RegNetwork human curated database using ChIP-Seq datasets as gold standards.

TF (GSE Acc #)	NTF	RegNetwork
Cebpa (GSM427088)	0.49473	0.245421
Cebpa (GSM427093)	0.33641	0.130647
E2f4 (GSM427091)	0.8174	0.837838
E2f4 (GSM427094)	0.90689	0.918919
Foxa1 (GSM427090)	0.11119	0
Foxa2 (GSM427089)	0.21049	0.157895

Table S4. Comparison of precision values between NTF and RegNetwork human curated database using TF knockout microarray datasets as gold standards.

TF (GSE Acc #)	NTF	RegNetwork
Atoh1	0.33073	0.428571
Pax6	0.37957	0.084848
Otx2 (GSE21900)	0.36028	0.111111
Otx2 (GSE27630)	0.41637	0

1.2 Supplementary Figures

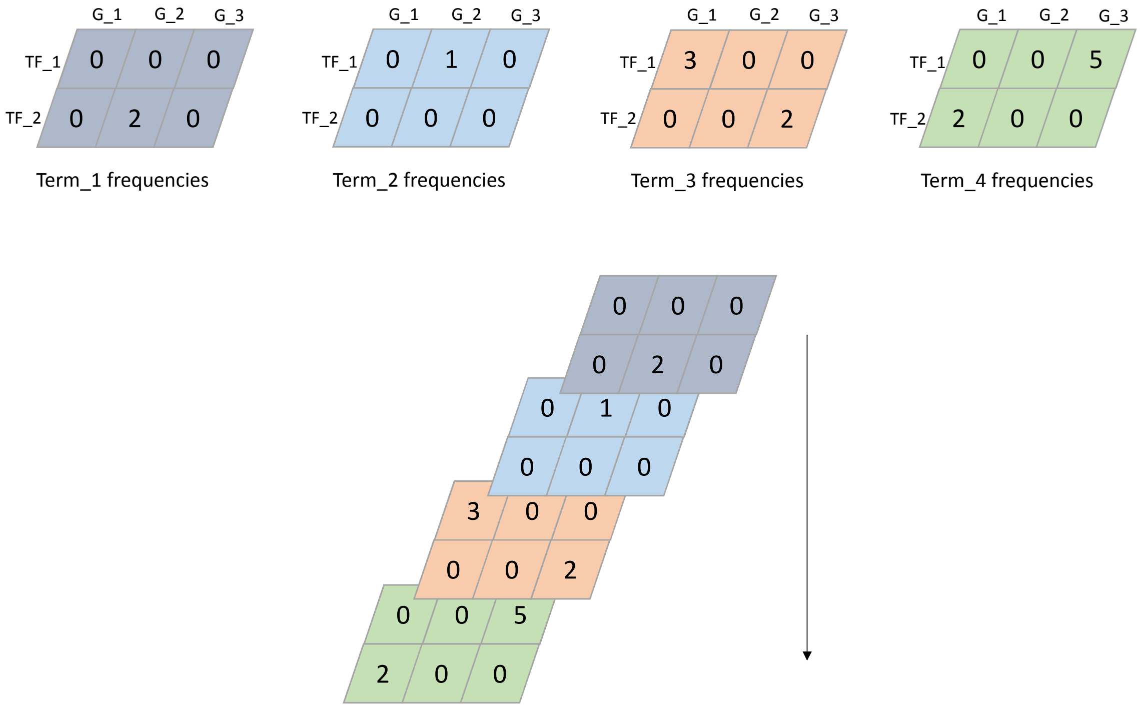

Figure S1. A toy example showing tensor construction for 4 terms (Term_1, Term_2, . . . , Term_4), 3 genes (G_1, G_2, G_3), and 2 TFs (TF_1, TF_2). For each term, the frequency of its occurrence in abstracts shared by each gene-TF pair is determined. These frequencies are stored in a matrix of size $\#Genes \times \#TFs$. Once a matrix is created for each term, all matrices can be layered vertically together to form a tensor of size $\#Terms \times \#Genes \times \#TFs$.

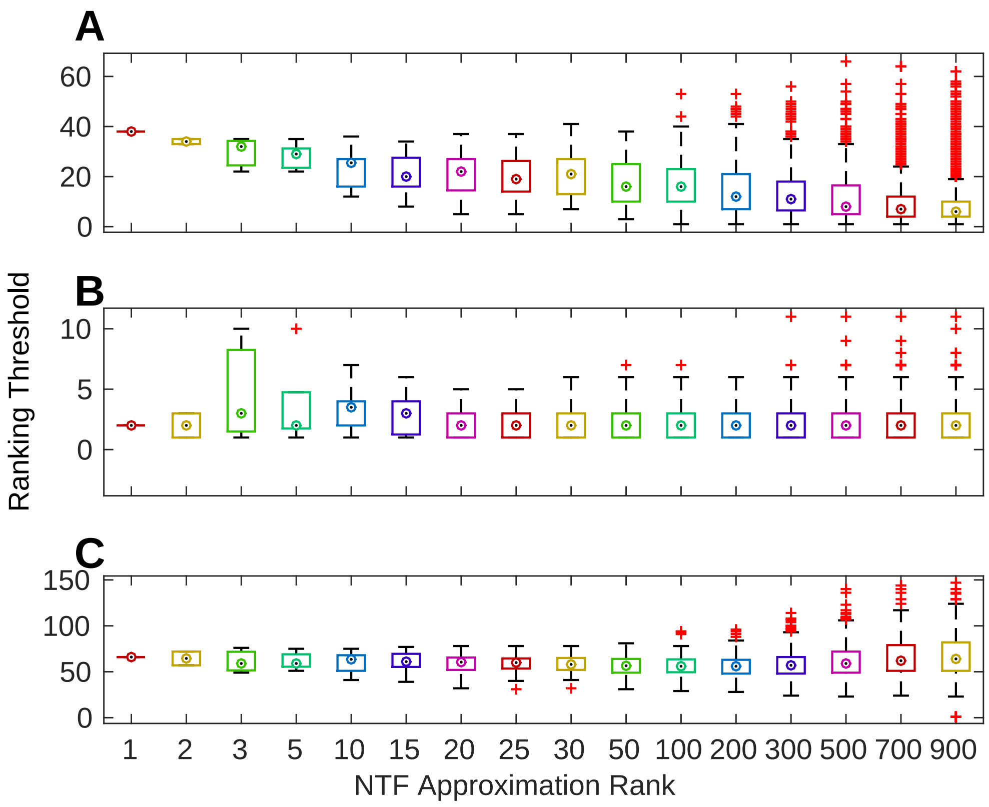

Figure S2. Distribution of numbers of genes (A), TFs (B) and terms (C) in the ATMs in various k-factorizations.

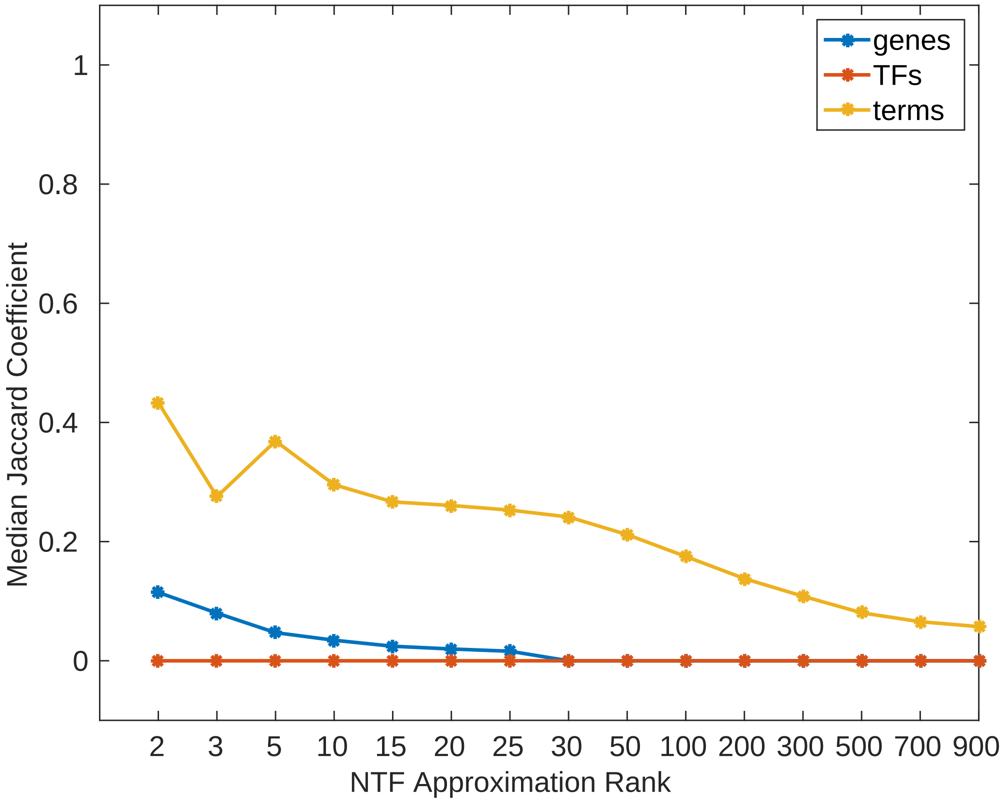

Figure S3. Redundancy between the terms, genes and TFs in the ATMs across various k-factorizations.

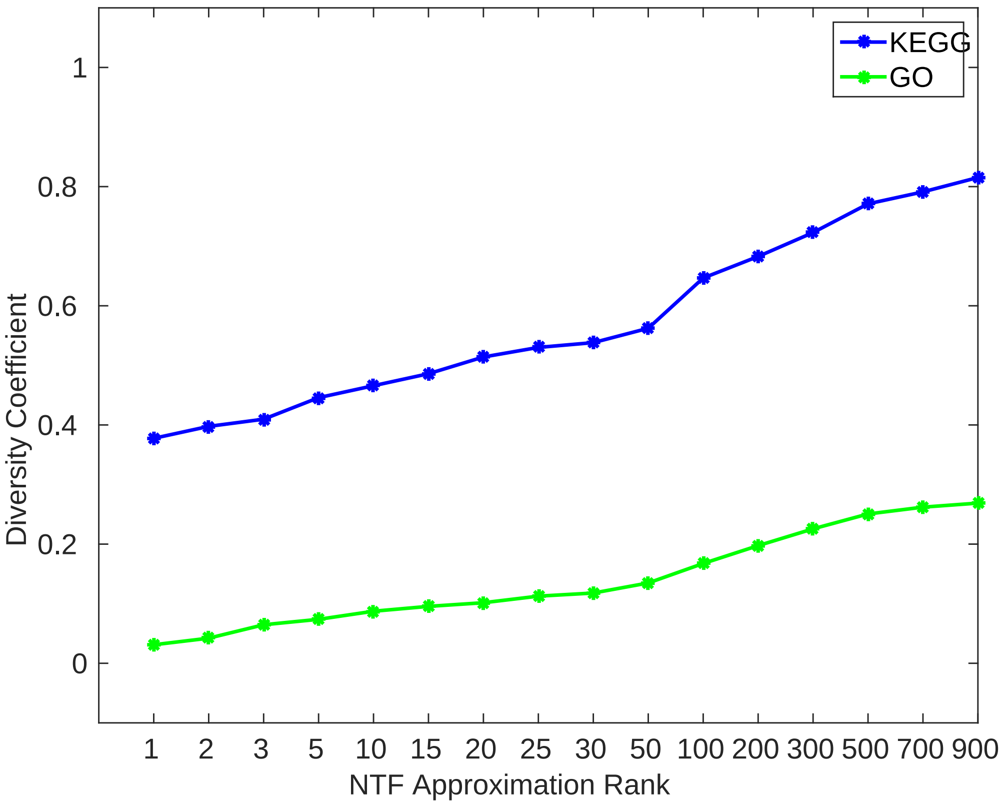

Figure S4. Diversity coefficients of the ATMs in terms of enrichment in KEGG pathways and GO categories in the various k-factorizations.

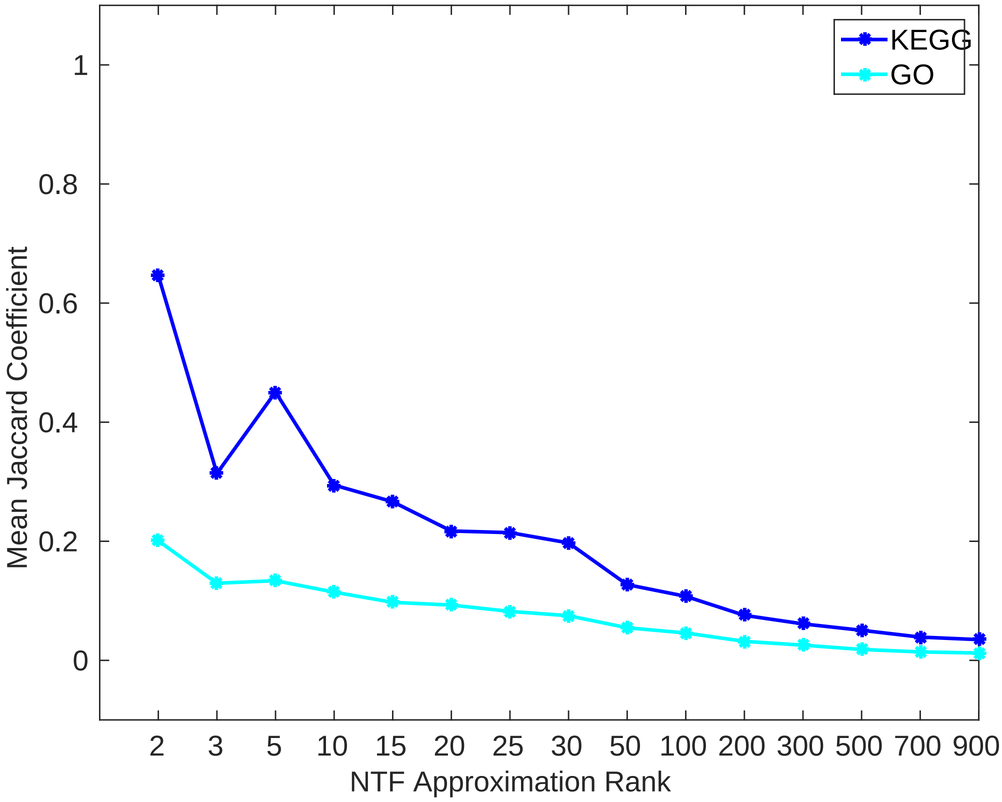

Figure S5. Redundancy between the ATMs in terms of enrichment in KEGG pathways and GO categories in the various k-factorizations.
